# Clinical impact of weight loss on mortality in patients with idiopathic pulmonary fibrosis: a retrospective cohort study

**DOI:** 10.1038/s41598-023-32843-7

**Published:** 2023-04-08

**Authors:** Ju Kwang Lee, Chiwook Chung, Jiwon Kim, Hyo Sin Cho, Ho Cheol Kim

**Affiliations:** 1 Department of Internal Medicine, Asan Medical Center, University of Ulsan College of Medicine, Seoul, Republic of Korea; 2 Department of Pulmonary and Critical Care Medicine, Asan Medical Center, University of Ulsan College of Medicine, 88 Olympic-ro 43-gil, Songpa-gu, Seoul, 05505 Republic of Korea; 3 University of Ulsan College of Medicine, Seoul, Republic of Korea

**Keywords:** Diseases, Medical research, Signs and symptoms

## Abstract

Patients with idiopathic pulmonary fibrosis (IPF) often experience weight loss during the follow-up period. However, the prevalence and clinical impact of weight loss in these patients still need to be elucidated. This retrospective single-center study reviewed 134 consecutive patients diagnosed with IPF. Weight loss of 5% or more over 1 year was defined as significant weight loss. Clinical data of patients were compared according to the significant weight loss. We analyzed whether the clinical impact of significant weight loss differed regarding the pirfenidone dose. The median follow-up period was 22.1 months. The mean age of patients was 67.3 years, and 92.5% were men. Of the 134 patients, 42 (31.3%) showed significant weight loss. Multivariate cox regression analysis revealed that significant weight loss was independently associated with mortality (hazard ratio [HR]; 2.670; 95% confidence interval [CI] 1.099–6.484; *p* = 0.030) after adjusting for lung function and other significant risk factors (6-min walk test distance: HR, 0.993; 95% CI 0.987–0.998; *p* = 0.005). The median survival of patients with significant weight loss (n = 22) was relevantly shorter than that of those without significant weight loss (n = 43) in the reduced dose pirfenidone group (28.2 ± 3.3 vs. 43.3 ± 3.2 months, *p* = 0.013). Compared with patients without significant weight loss (n = 38), patients with significant weight loss (n = 15) also showed a marginally-significant shorter survival in the full-dose pirfenidone group (28.9 ± 3.1 vs. 39.8 ± 2.6 months, *p* = 0.085). Significant weight loss is a prognostic factor in patients with IPF regardless of pirfenidone dose. Vigilant monitoring might be necessary to detect weight loss during the clinical course in these patients.

## Introduction

Idiopathic pulmonary fibrosis(IPF) is a specific form of interstitial lung disease characterized by chronic and progressive loss of lung function^1^. Although the clinical course of IPF varies widely, in general, the prognosis of IPF is poor, and the median survival is 3–5 years from the time of diagnosis^2^.

Weight loss is a common problem in patients with IPF. Previous studies have reported that approximately 15–20% of patients with IPF lose $\ge$ 5% of their weight during the first year of the disease course^3,4^. Various factors such as disease progression, chronic inflammation, and physical inactivity have been suggested as the cause of weight loss in patients with IPF^5–7^. In addition, anti-fibrotic medications to treat IPF, especially pirfenidone might cause weight loss^8^, often resulting in a dose reduction of these medications to minimize adverse effects^9^.

Several studies have reported that weight loss and low body mass index (BMI) can adversely affect the prognosis of patients with IPF^3,4,10,11^. However, other studies have shown conflicting results, depending on the confounding factor and study design^12,13^. Large-scale studies on the prevalence and clinical impact of weight loss in patients with IPF are still needed, especially in the era of anti-fibrotic medication usage, which is an important confounding factor. Therefore, our study aimed to investigate the prevalence, risk factors, clinical impact of weight loss in patients with IPF, and whether the clinical effects of weight loss differ in regard to the dose of pirfenidone.

## Methods

### Study population

In this single-center retrospective cohort study, we analyzed the data of patients aged $\ge$ 40 years and diagnosed with IPF at Asan Medical Center, a tertiary referral hospital in Seoul, South Korea between January 1, 2018, and September 1, 2021. Patients with a follow-up duration of less than a year, and patients with no data on body weight at the time of diagnosis or a year after diagnosis were excluded from the study. Patients with a definite connective tissue disease or a history of exposure to the possible causes of interstitial lung disease were also excluded.

Diagnosis of IPF was made by the diagnostic criteria of the American Thoracic Society (ATS)/European Respiratory Society (ERS)/Japanese Respiratory Society/Latin American Thoracic Association in 2018^14^.

### Clinical data

Body weight was measured at the time of diagnosis and the outpatient visit or hospitalization thereafter. Significant weight loss was defined as a weight loss of $\ge$ 5% over 1 year after diagnosis^15,16^. Data from the National Insurance Corporation were used to investigate deaths and their dates from the time of diagnosis to August 1, 2022. Consistent with ERS/ATS recommendations, spirometry was performed and, total lung capacity (TLC), and diffusing capacity for carbon monoxide (DLco) were measured^17,18^. The 6-min walk test (6MWT) was also performed according to these guidelines^19^. We used in-hospital data to investigate the demographics of the patients, whether they used pirfenidone, the dosage and side effects of pirfenidone, and the types of side effects. We defined the use of pirfenidone as a prescription with pirfenidone for more than 3 months. As used in Korea, full dose of pirfenidone was defined as 1800 mg/d, and reduced dose of pirfenindone as < 1800 mg/d^8,20^.

### Statistical analyses

For continuous variables, all values are presented as means ± standard deviations and categorical variables are presented as percentages. Student’s *t* test or Mann–Whitney test was used to compare continuous variables, and the χ^2^ test or Fisher’s exact test was used to compare categorical variables.

The patients were divided into two groups: one with significant weight loss and one without. Logistic analysis was used to find risk factors for significant weight loss. Factors affecting mortality in patients with IPF were analyzed through the Cox proportional hazard analysis. Variables significant at a *p* value of $\le$ 0.2 in the univariable analysis were candidates in the multivariable analysis. Survival analysis was performed to determine a difference in mortality between the two groups using the Kaplan–Meier survival analysis and log-rank test.

All *p*-values were two-tailed, with statistical significance set at *p* $\le$ 0.05. SPSS 22.0 software (IBM Corporation, Armonk, NY, USA) was used for all statistical analyses.

### Ethics approval and consent to participate

The study protocol was endorsed by the Institutional Review Board of Asan Medical Center (IRB number: 2021-0736). This study was conducted in compliance with the Declaration of Helsinki. Informed consent was waived because of the retrospective study design and anonymity of clinical data.

## Results

### Patients

A total of 134 patients were included in this study. The median follow-up duration was 22.1 months (interquartile range: 15.0–29.4 months). Table 1 presents the baseline characteristics of patients according to significant weight loss. Most participants were male (92.5%) and ever-smokers (86.6%). Of the total patients, 42 (31.3%) had a significant weight loss during the follow-up period, and 92 (68.7%) did not.

**Table 1:** Patient characteristics.

	Total	Group with significant weight loss	Group without significant weight loss	*P* value
Patients, n	134	42	92	
Age, years	67.3 ± 8.2	67.3 ± 9.3	67.3 ± 7.8	0.985
Male sex	124 (92.5)	41 (97.6)	83 (90.2)	0.171
Ever-smokers	116 (86.6)	39 (92.9)	77 (83.7)	0.149
Baseline body weight, kg	69.2 ± 10.6	70.4 ± 11.2	68.6 ± 10.3	0.366
Baseline BMI, kg/m^2^	25.5 ± 3.06	25.9 ± 3.37	25.3 ± 2.90	0.294
Weight change, kg/year	− 2.14 ± 4.99	− 7.83 ± 3.81	0.45 ± 2.84	< 0.001
IPF diagnosis				
Clinical IPF	103 (76.9)	31 (73.8)	78.3	0.571
Biopsy proven IPF	31 (23.1)	11 (26.2)	21.7	
KL-6, U/mL (116, 36/80)	1014.4 ± 874.9	922.3 ± 692.2	1055.8 ± 951.9	0.452
Baseline albumin, g/dL (126, 40/86)	3.67 ± 0.43	3.58 ± 0.45	3.72 ± 0.41	0.108
Emphysema	62 (46.3)	17 (40.5)	45 (48.9)	0.364
Comorbidities				
COPD	12 (9.0)	2 (4.8)	10 (10.9)	0.339
Lung cancer	20 (14.9)	11 (26.2)	9 (9.8)	0.013
GERD	16 (11.9)	4 (9.5)	12 (13.0)	0.560
Ischemic heart disease	22 (16.4)	8 (19.0)	14 (15.2)	0.579
Pulmonary function test				
FVC, % predicted (129, 41/88)	75.6 ± 13.7	72.5 ± 15.1	77.0 ± 13.0	0.086
DLco, % predicted (124, 39/85)	58.6 ± 16.8	53.9 ± 16.1	60.7 ± 16.8	0.035
TLC, % predicted (112, 35/77)	74.5 ± 12.4	73.0 ± 13.3	75.2 ± 12.1	0.393
6MWD, meter	423.4 ± 99.9	414.0 ± 95.1	427.7 ± 102.8	0.468
6MWT, lowest SpO_2_, %	90.4 ± 5.8	89.7 ± 5.5	90.8 ± 6.0	0.356
GAP stage				
GAP stage I	62 (46.3)	16 (38.1)	46 (50.0)	0.193
GAP stage II	67 (50.0)	23 (54.8)	44 (47.8)	
GAP stage III	5 (3.7)	3 (7.1)	2 (2.2)	

Data presented as mean ± standard deviation, median (interquartile range), or n (%), unless otherwise indicated. If a variable could not be measured in all patients, the number of patients for whom the variable was measured in total population and each group is indicated in parentheses next to the variables in the first column.

*BMI* body mass index, *COPD* chronic pulmonary obstructive disease, *GERD* gastroesophageal reflux disease, *FVC* forced vital capacity, *DLco* diffusing capacity of the lung for carbon monoxide, *IPF* idiopathic pulmonary fibrosis, *TLC* total lung capacity, *6MWD* 6-min walk test distance, *6MWT* 6-min walk test, *SpO*_2_ peripheral oxygen saturation, *GAP* Gender, Age, Physiology.

Baseline characteristics were similar between the two groups; however, the DLco was significantly lower in the weight loss group (*p* = 0.035) than in the other group. Baseline body weight, BMI and GAP (Gender, Age, Physiology) class^21^ were not significantly different between two groups (*p* = 0.366, *p* = 0.294, and *p* = 0.193, respectively). Baseline Forced vital capacity (FVC) and albumin levels were lower in the significant weight loss group; however, the differences were marginally significant (FVC: *p* = 0.086, albumin: *p* = 0.108).

Patients with significant weight loss had an average weight loss of 7.83 kg over 1 year, and patients without significant weight loss had an average weight gain of 0.45 kg over 1 year (*p* < 0.001).

### Use of pirfenidone and associated side effects

Most patients (88.1%, 118 of 134) received pirfenidone, and no significant difference was observed between the two groups regarding the pirfenidone dose, duration*,* and discontinuation of pirfenidone. (*p* = 0.518, *p* = 0.257*, p* = 0.722 respectively, Table 2). The median time period from the diagnosis of IPF to initiation of pirfenidone was 2.1 months (interquartile range: 0.3–6.6 months) and showed no significant difference between the two groups (*p* = 0.213). Of all patients, 37.3% had gastrointestinal, 37.3% had skin, and 12.7% had both side effects. There was no significant difference in the frequency of each side effect according to significant weight loss (*p* > 0.999, *p* = 0.744, *p* = 0.774 for gastrointestinal, skin, and both side effects, respectively).

**Table 2:** Use of pirfenidone and associated side effects in subgroups of patients by weight loss.

	Total	Group with significant weight loss	Group without significant weight loss	*P* value
Patients, n	118	37	81	
Pirfenidone full dose	53 (44.9)	15 (40.5)	38 (46.9)	0.518
Pirfenidone reduced dose	65 (55.1)	22 (59.5)	43 (53.1)	
Duration of pirfenidone therapy (months)	17.6 ± 9.4	16.1 ± 9.8	18.2 ± 9.1	0.257
Diagnosis to pirfenidone therapy (months)	2.1 (0.3–6.6)	2.0 (0.2–4.1)	2.2 (0.4–7.7)	0.213
Discontinuation of treatment	18 (15.3)	5 (13.5)	13 (16.0)	0.722
Side effect				
GI	44 (37.3)	14 (37.8)	30 (37.0)	> 0.999
Skin	44 (37.3)	13 (35.1)	31 (38.3)	0.744
GI and skin	15 (12.7)	4 (10.8)	11 (13.6)	0.774

Data presented as mean ± standard deviation, n (%), median (interquartile range).

*GI* gastrointestinal.

### Risk factors related to significant weight loss

No baseline characteristics including age, sex, smoking history, BMI, baseline albumin, emphysema, and 6MWT showed a relevant relation with significant weight loss, except comorbidity of lung cancer (odds ratio [OR] = 2.828, *p* = 0.047). Although DLco was identified as a statistically relevant risk factor for significant weight loss (odds ratio [OR] = 0.975, *p* = 0.038) in the univariate analysis, the multivariate analysis showed no statistical significance (OR = 0.984, *p* = 0.256, Table 3).

**Table 3:** Logistic regression analysis of risk factors for weight loss.

Variable	OR	95% CI	*P* value
Univariate analysis			
Age, years	1.000	0.956–1.045	0.985
Male sex	4.446	0.545–36.290	0.164
Ever smokers	2.532	0.692–9.274	0.161
Baseline BMI, kg/m^2^	1.065	0.947–1.199	0.294
Baseline albumin, g/dL	0.496	0.208–1.182	0.113
Emphysema	0.710	0.339–1.488	0.364
COPD	0.410	0.086–1.960	0.264
Lung cancer	3.272	1.237–8.656	0.017
GERD	0.702	0.212–2.320	2.320
Ischemic heart disease	1.311	0.503–3.415	0.579
FVC, % predicted	0.975	0.948–1.004	0.089
DLco, % predicted	0.975	0.952–0.999	0.038
TLC, % predicted	0.986	0.954–1.018	0.389
6MWD, meter	0.999	0.995–1.002	0.465
6MWT, lowest SpO_2_, %	0.972	0.914–1.033	0.355
Multivariate analysis			
Male sex	1.842	0.083–40.976	0.699
Ever smokers	1.947	0.208–18.200	0.559
Baseline albumin, g/dL	0.561	0.208–1.516	0.255
Lung cancer	2.828	1.015–7.877	0.047
FVC, % predicted	0.988	0.956–1.020	0.444
DLco, % predicted	0.984	0.958–1.011	0.256

*BMI* body mass index, *COPD* chronic pulmonary obstructive disease, *GERD* gastroesophageal reflux disease, *FVC* forced vital capacity, *DLco* diffusing capacity of the lung for carbon monoxide, *IPF* idiopathic pulmonary fibrosis, *TLC* total lung capacity, *6MWD* 6-min walk test distance, *6MWT* 6-min walk test, *SpO*_2_ peripheral oxygen saturation.

### Factors affecting mortality and survival analysis

Table 4 demonstrates the Cox regression analysis of risk factors for mortality. The univariate Cox analysis showed that significant weight loss (hazard ratio [HR], 3.208; 95% confidence interval [CI] 1.452–7.089; *p* = 0.004), baseline albumin (HR, 0.394; 95% CI 0.199–0.781; *p* = 0.008), FVC (HR, 0.974; 95% CI 0.945–1.003; *p* = 0.075), DLco (HR, 0.954; 95% CI 0.928–0.980; *p* < 0.001), 6-min walking distance [6MWD] HR, 0.993; 95% CI 0.990–0.997; *p* < 0.001), lowest oxygen saturation (SpO_2_) in 6MWT (HR, 0.928; 95% CI 0.879–0.979; *p* = 0.006), and use of pirfenidone (HR, 0.367; 95% CI 0.145–0.931; *p* = 0.035) were associated with mortality in patients with IPF. Although FVC and DLco were significantly associated with mortality in patients with IPF in univariate analysis, GAP stage was not significantly associated with mortality (Supplementary Table 1).

**Table 4:** Cox regression analysis of risk factors for mortality.

Variable	HR	95% CI	*P* value
Univariate analysis			
Significant weight loss	3.208	1.452–7.089	0.004
Age, years	0.997	0.952–1.043	0.890
Male sex	0.970	0.288–3.267	0.961
Ever smokers	1.739	0.520–5.816	0.369
Baseline BMI, kg/m^2^	0.938	0.818–1.075	0.357
Baseline albumin, g/dL	0.394	0.199–0.781	0.008
COPD	0.039	0.000–6.822	0.218
Lung cancer	1.886	0.639–5.563	0.250
GERD	0.476	0.112–2.024	0.315
Ischemic heart disease	2.042	0.846–4.930	0.112
Pulmonary function test			
FVC, % predicted	0.974	0.945–1.003	0.075
DLco, % predicted	0.954	0.928–0.980	< 0.001
TLC, % predicted	0.974	0.944–1.005	0.101
6MWD, meter	0.993	0.990–0.997	< 0.001
6MWT, lowest SpO_2_, %	0.928	0.879–0.979	0.006
Use of pirfenidone	0.367	0.145–0.931	0.035
Multivariate analysis			
Significant weight loss	2.670	1.099–6.484	0.030
Baseline albumin, g/dL	0.499	0.207–1.201	0.121
Ischemic heart disease	1.389	0.487–3.962	0.538
FVC, % predicted	0.984	0.953–1.017	0.338
DLco, % predicted	0.974	0.940–1.009	0.139
6MWD, meter	0.993	0.987–0.998	0.005
6MWT, lowest SpO_2_, %	1.005	0.918–1.099	0.920
Use of pirfenidone	0.582	0.190–1.781	0.342

Among the significant variables in the univariate analysis, TLC (r = 0.974, *p* = 0.101) was excluded in the multivariate analysis due to close correlation with FVC.

*HR* hazard ratio, *CI* confidence interval, *BMI* body mass index, *COPD* chronic pulmonary obstructive disease, *GERD* gastroesophageal reflux disease, *FVC* forced vital capacity, *DLco* diffusing capacity of the lung for carbon monoxide, *IPF* idiopathic pulmonary fibrosis, *TLC* total lung capacity, *6MWD* 6-min walk test distance, *6MWT* 6-min walk test, *SpO*_2_ peripheral oxygen saturation.

**Figure 1:**
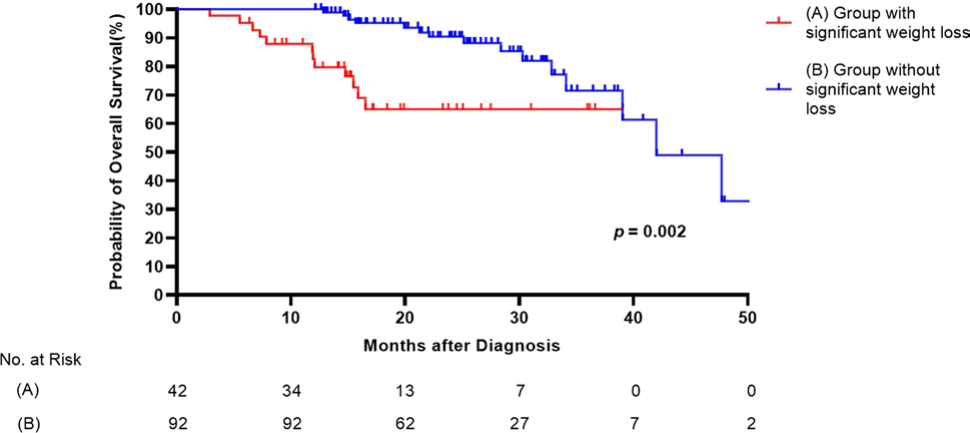
Comparison of survival curves between groups and without significant weight loss among patients with IPF. (A) Group with significant weight loss, (B) Group without significant weight loss.

In the multivariate analysis, significant weight loss was independently associated with mortality (HR, 2.670; 95% CI 1.099–6.484; *p* = 0.030) after adjusting other risk factors. And 6MWD was also an independent risk factor of mortality (6MWD: HR, 0.993; 95% CI 0.987–0.998; *p* = 0.005). A comparison of survival curves between the patients with and without weight loss is shown in Fig. 1. The median survival of patients with weight loss was shorter than that of those without weight loss (29.4 ± 2.3 vs. 41.4 ± 2.1 months, respectively, *p* = 0.002).

### Clinical impact of weight loss according to the pirfenidone dosage

**Figure 2:**
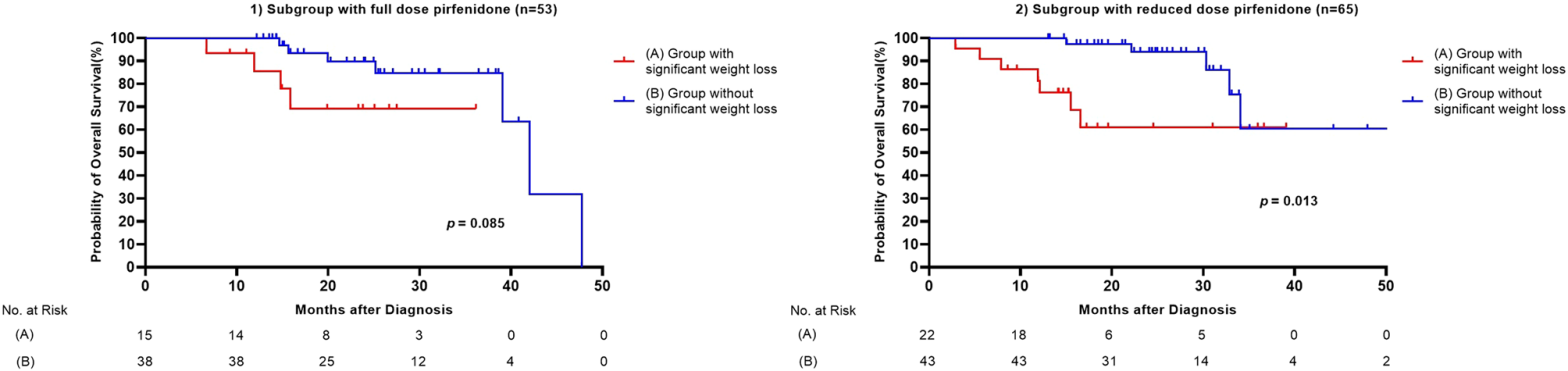
(**1**) Comparison of survival curves between groups with and without significant weight loss in a subgroup with full-dose pirfenidone (A) Group with significant weight loss, (B) Group without significant weight loss. (**2**) Comparison of survival curves between groups with and without significant weight loss in a subgroup with reduced dose pirfenidone (A) Group with significant weight loss, (B) Group without significant weight loss.

We analyzed whether the effect of significant weight loss on mortality differed according to the pirfenidone dose. The 118 patients treated with pirfenidone were divided into full-dose (1800 mg/d, 53 patients) and reduced-dose groups ($<$ 1800 mg/d, 65 patients). Survival analysis was performed, and a Kaplan–Meier curve was plotted (Fig. 21,2). The median survival of patients with relevant weight loss was significantly shorter than that of those without significant weight loss in the reduced dose pirfenidone group (28.2 ± 3.3 vs. 43.3 ± 3.2 months, *p* = 0.013). In the full-dose group, the median survival was tended to be shorter in the weight loss group (28.9 ± 3.1 vs. 39.8 ± 2.6 months, *p* = 0.085).

## Discussion

In this study, 42 of 134 (31.3%) patients showed significant weight loss during the follow-up period. There were no differences in the usage and dosage of pirfenidone between the groups with and without significant weight loss. Significant weight loss had an independent effect on mortality even after adjusting for lung function and exercise capacity, and the subgroup analysis according to the pirfenidone dose showed similar results.

Although the exact mechanism of these findings needs to be clarified, oxidative stress, chronic inflammation, physical inactivity, and poor general condition are proposed causes of weight loss in patients with IPF^5,6,22,23^. In this study, proportion of patients who had significant weight loss (31.3%) was higher than that of previous studies^3,10^. Nakatsuka et al. reported that the prevalence of significant weight loss in patients with IPF was 21.8% (27 of 124) in the Japanese cohort and 15.1% (13 of 86) in the English cohort^3^. Similarly, a retrospective cohort study by Jouneau et al. showed that 20.1% (85 of 423) of patients with IPF who took a placebo rather than nintedanib showed significant weight loss^10^. Interestingly, our current study showed that baseline low DLco was a risk factor for significant weight loss, though its association was not significant in multivariate analysis. The exact mechanism by which the decrease in DLco becomes the risk factor for weight loss is unclear, but a possible explanation is that emphysema or pulmonary hypertension often accompanies a decrease in DLco, and they could be causes of weight loss. Previous studies reported COPD, including emphysema could accompany weight loss^24,25^. Another reported that pulmonary hypertension also impairs functional status and quality of life^26^”. The following factors likely contributed to a higher proportion of patients having significant weight loss in our study compared with previous studies: first, in the general population, Asians have a lower body weight than Caucasians^27^; second, the number of patients who took pirfenidone in our study was higher than that of the previous study (88.1% of patients in our study took pirfenidone; 28.2% and 23.3% took pirfenidone in the Japanese and English cohorts by Nakatsuka et al. respectively; and 60.1% took nintedanib in the study by Jouneau et al.); and finally, the exclusion of patients who followed-up for $\le$ 1 year from the study design might have affected the prevalence. Among patients with IPF, Asians also have a higher proportion of underweight patients than do Caucasians, suggesting regional and racial differences in the clinical course of IPF^10,28^.

Several previous studies have shown that weight loss might be associated with poor prognosis in patients with IPF. In Nakatsuka et al.’s retrospective cohort study of 210 patients, a weight loss of $\ge$ 5% was a significant risk factor for mortality (HR = 2.51, 95% CI 1.01–6.23, *p* = 0.047). Weight loss suggested a worse outcome even when FVC was normal^3^. Pugashetti et al. in a study with 225 patients with interstitial lung disease, reported that every 1% yearly decrease in BMI was associated with a 5% increase in mortality risk^4^. Furthermore, a recent report revealed that malnutrition is common in patients with IPF and associated with hospitalization and mortality, supporting the inference that weight loss adversely affects the prognosis of patients with IPF^29^.

Similar to the previous findings, significant weight loss was associated with poor outcomes in our study. It was associated with a poor prognosis even after adjusting for well-known prognostic factors such as 6MWD, baseline albumin levels, and lung function^1,30,31^. Therefore, it can be suggested that careful monitoring of weight loss is necessary when treating patients with IPF. Additionally, the GAP stage, which showed significant predictive ability for mortality in IPF patients^21,32,33^, was reported to be not associated in accurately predicting the prognosis in East Asia^34,35^. In this study, the GAP stage was not associated with mortality, either.

Although anorexia and weight loss are known side effects of pirfenidone^7^, there was no significant difference in the pirfenidone dose and the frequency of side effects between the significant and non-significant weight loss groups in our study. Pirfenidone therapy was started a median of 2.1 months after diagnosis of IPF and was administered for an average of 17.6 months. There was no significant difference in the time from diagnosis to the start of pirfenidone therapy and the duration of pirfenidone therapy according to weight loss.

Although the median survival was tended to be shorter in the weight loss group among patients who received full dose pirfenidone, the median survival of patients with relevant weight loss was significantly shorter in the reduced dose pirfenidone group. These finding suggest that weight loss might be still an important prognostic factor in patients with IPF using pirfenidone. Similarly, Jouneau et al. conducted a post hoc analysis of previous pirfenidone-related studies and suggested that patients whose BMI is $\le$ 25 $\mathrm{kg}/{\mathrm{m}}^{2}$ or who experienced weight loss in the placebo group showed a faster rate of FVC decline and worse prognosis^36^. Similarly, in the pirfenidone-treated group, patients with significant weight loss showed a worse prognosis than did those without significant weight loss^36^. However, nintedanib, another anti-fibrotic agent, showed different results in a previous study^10^. Although in the placebo group, the rate of FVC decline was faster in patients with a baseline BMI $\le$ 25 $\mathrm{kg}/{\mathrm{m}}^{2}$ than in patients with a BMI of 25 $\mathrm{kg}/{\mathrm{m}}^{2}$ or more, FVC changes between each BMI group were similar in the nintedanib group^10^. Likewise, patients with significant weight loss suffered a relevantly faster decrease in FVC in the placebo group; however, the rate of FVC decline was not significantly different depending on significant weight loss in the nintedanib group^10^. Although some conflicting results have been reported between the two anti-fibrotic agents, weight loss might be an important composite outcome in patients with IPF who receive anti-fibrotic agents.

This study had several limitations. First, this was a single-center and retrospective study. Due to the retrospective design, we could not include all variables that could potentially affect mortality. In addition, as the retrospective design of the study, there might be some selection bias. In our current study, a total of 92.5% of study population were male patients, which was extremely higher compared to the other previous studies. However, among patients with probable usual interstitial pneumonia pattern, only old male with smoking history were considered as clinical IPF, for diagnostic certainty. Second, we could not specifically investigate the nutritional status and quality of life of the patients. However, we measured serum albumin level and used it as a surrogate for nutrition status. Third, in Korea, nintedanib is rarely used because it is not covered by health insurance; therefore, a detailed analysis of nintedanib could not be conducted in this study. And most of the patients included in this study took pirfenidone; therefore, we could not analyze the effect of weight loss on patients with IPF who did not take pirfenidone. Lastly, in Figs. 1 and 2, the survival difference between the two groups almost disappears at 30–40 months after diagnosis. In this study, the patient's median follow-up duration was 21.2 months (IQR 15.0–28.3). The insufficient amount of long-term follow-up patients might cause obscuration the survival difference between the two groups after 30–40 months. Further study with a longer duration is needed.

## Conclusions

Our study suggests that significant weight loss might be an important prognostic factor in patients with IPF. Therefore, a vigilant monitoring is required to detect weight loss during the clinical course in patients with IPF.

## Supplementary Information

Supplementary Table 1.

## Data Availability

The datasets generated and/or analyzed during this study are available from the corresponding author on reasonable request.

## Abbreviations

IPF: Idiopathic pulmonary fibrosis
FVC: Forced vital capacity
DLco: Diffusing capacity for carbon monoxide
TLC: Total lung capacity
PFT: Pulmonary function test
BMI: Body mass index
6MWD: 6-minute walk test distance
6MWT: 6-minute walk test
SpO2: Peripheral oxygen saturation
GI: Gastrointestinal
HR: Hazard ratio
CI: Confidence interval
OR: Odds ratio
