# Supplementary material for: Clinical impact of weight loss on mortality in patients with idiopathic pulmonary fibrosis: a retrospective cohort study
Source: Sci Rep. 2023 Apr 8;13:5774. doi: 10.1038/s41598-023-32843-7 (PMC10082839; doi:10.1038/s41598-023-32843-7)
Supplement: Supplementary file 1 — Supplementary Table 1. [file 41598_2023_32843_MOESM1_ESM.docx]

**Supplementary table 1.** Cox regression analysis of risk factors for mortality, with GAP stage

| Variable | HR | 95% CI | *p* value |
| --- | --- | --- | --- |
| Univariate analysis | | | |
| Significant weight loss | 3.208 | 1.452-7.089 | 0.004 |
| Ever smokers | 1.739 | 0.520-5.816 | 0.369 |
| Baseline BMI, kg/m^2^ | 0.938 | 0.818-1.075 | 0.357 |
| Baseline albumin, g/dL | 0.394 | 0.199-0.781 | 0.008 |
| COPD | 0.039 | 0.000-6.822 | 0.218 |
| Lung cancer | 1.886 | 0.639-5.563 | 0.250 |
| GERD | 0.476 | 0.112-2.024 | 0.315 |
| Ischemic heart disease | 2.042 | 0.846-4.930 | 0.112 |
| GAP stage |  |  |  |
| GAP stage I | reference |  |  |
| GAP stage II | 1.209 | 0.555-2.635 | 0.632 |
| GAP stage III | 2.442 | 0.310-19.253 | 0.397 |
| 6MWD, meter | 0.993 | 0.990-0.997 | < 0.001 |
| 6MWT, lowest SpO_2_, % | 0.928 | 0.879-0.979 | 0.006 |
| Use of pirfenidone | 0.367 | 0.145-0.931 | 0.035 |
| Multivariate analysis | | | |
| Significant weight loss | 3.172 | 1.346-7.476 | 0.008 |
| Baseline albumin, g/dL | 0.425 | 0.193-0.935 | 0.033 |
| Ischemic heart disease | 2.106 | 0.797-5.567 | 0.133 |
| 6MWD, meter | 0.994 | 0.990-0.998 | 0.005 |
| 6MWT, lowest SpO_2_, % | 0.963 | 0.895-1.037 | 0.317 |
| Use of pirfenidone | 0.551 | 0.189-1.605 | 0.274 |

Among the significant variables in the univariate analysis, TLC (r = 0.974, *p* = 0.101) was excluded in the multivariate analysis due to close correlation with FVC. HR: hazard ratio; CI: confidence interval; BMI: body mass index; COPD: chronic pulmonary obstructive disease; GERD: gastroesophageal reflux disease; FVC: forced vital capacity; DLco: diffusing capacity of the lung for carbon monoxide; IPF: idiopathic pulmonary fibrosis; TLC: total lung capacity; 6MWD: 6-minute walk test distance; 6MWT: 6-minute walk test; SpO_2_: peripheral oxygen saturation; GAP: Gender, Age, Physiology.
